# Diagnostic accuracy of computer aided reading of chest x-ray in screening for pulmonary tuberculosis in comparison with Gene-Xpert

**DOI:** 10.12669/pjms.38.1.4531

**Published:** 2022

**Authors:** Tahira Nishtar, Shamsullah Burki, Fatima Sultan Ahmad, Tabish Ahmad

**Affiliations:** 1 Tahira Nishtar FCPS, Department of Radiology, MTI-Lady Reading Hospital, Peshawar, Pakistan.; 2 Shamsullah Burki FCPS, Department of Radiology, MTI-Lady Reading Hospital, Peshawar, Pakistan; 3 Fatima Sultan Ahmad (Registrar), Department of Radiology, MTI-Lady Reading Hospital, Peshawar, Pakistan; 4 Tabish Ahmad (PGR-FCPS) Department of Radiology, MTI-Lady Reading Hospital, Peshawar, Pakistan

**Keywords:** Pulmonary tuberculosis, Computer-aided detection tuberculosis software, GeneXpert, Chest X-ray, Screening

## Abstract

**Background & Objectives:**

Pakistan ranked fifth amongst 22 high-burden Tuberculosis countries, and it is an epidemic in Pakistan, hence screening is performed nationally, as part of the ambitious ZERO TB drive. Our objective was to assess the diagnostic accuracy of Computer Aided Detection (CAD4TB) software on chest Xray in screening for pulmonary tuberculosis in comparison with gene-Xpert.

**Methods:**

The study was conducted by Radiology Department Lady Reading Hospital Peshawar in affiliation with Indus Hospital network over a period of one year. Screening was done by using mobile Xray unit equipped with CAD4TB software with scoring system. All of those having score of more than 70 and few selected cases with strong clinical suspicion but score of less than 70 were referred to dedicated TB clinic for Gene-Xpert analysis.

**Results:**

Among 26,997 individuals screened, 2617 (9.7%) individuals were found presumptive for pulmonary TB. Sputum samples for Gene-Xpert were obtained in 2100 (80.24%) individuals, out of which 1825 (86.9%) were presumptive for pulmonary TB on CAD4TB only. Gene-Xpert was positive in 159 (8.7%) patients and negative in 1,666(91.3%). Sensitivity and specificity of CAD4TB and symptomatology with threshold score of ≥70 was 83.2% and 12.7% respectively keeping Gene-Xpert as gold standard.

**Conclusion:**

Combination of chest X-ray analysis by CAD4TB and symptomatology is of immense value to screen a large population at risk in a developing high burden country. It is significantly a more effective tool for screening and early diagnosis of TB in individuals, who would otherwise go undiagnosed.

**Abbreviations:**
TB =: Tuberculosis
WHO =: World Health Organization
CAD4TB =: Computer aided detection for tuberculosis
CXR =: Chest X-Ray
CAR =: Computer aided reading

## INTRODUCTION

Tuberculosis (TB) is the most common disease caused by a solitary infectious agent. It is one of the top 10 causes of death globally. TB exists in all countries and age groups despite being a preventable and curable disease. About 10 million people were infected globally in 2019 including 5.6 million men, 3.2 million women and 1.2 million children.^1^ Pakistan has been ranked fifth among 22 high-burden TB countries of the world and accounts for 61% of the TB burden in the World Health Organization (WHO) Eastern Mediterranean region. The WHO estimated 510,000 new TB cases developing annually which has become an emerging health issue with great challenges.^2^

In 2016, as part of the ambitious ZERO TB drive, a private organization Indus Health Network initiated screening for early diagnosis of TB nationally by using mobile X-ray unit equipped with computer aided detection for tuberculosis (CAD4TB) software as a screening tool prior to laboratory testing.^3^

Pulmonary Tuberculosis is frequently diagnosed on clinical and radiological findings.^4^ TB screening for active case finding is an essential tool towards developing high quality TB control services. The goal of screening is early detection and treatment of disease. Systematic screening for active pulmonary TB is defined as the systematic identification of people with suspected active TB in a predetermined target group, using tests, examinations or other procedures that can be applied rapidly.^5^

However, TB screening outside hospitals requires screening a very large population who do not have TB resulting in high costs. Research suggests that chest X-Ray (CXR) is a primary screening tool for identifying persons in the population who require additional investigations for final diagnosis of tuberculosis.^6^ To ensure a satisfactory and accurate screening program, one needs to overcome limitations in the form of the cost of the diagnostic tests both radiological and laboratory, along with availability of trained supporting staff for a fast turnover of CXR image analysis. This led to development of an automated screening system for chest radiography in the form of computer aided reading (CAR). CAD4TB is a software in which various subsystems for the detection of textural and shape abnormalities, for symmetry and correlation analysis are used at pixel and image level.^7^ The software can evaluate digital radiographs by picking up abnormalities in the lung fields, hence identifying those people in the population who need to be investigated further. CAR software for TB evaluation is the need of the time in high TB burden population. The software has a scoring system and a cutoff score, above which additional TB testing referral is done. Literature evaluating the software sensitivity suggests that its performance is equivalent to human readers.^8^ Triage tests in the form of CXR as a screening tool and GeneXpert assay are the backbone of an accurate screening program. The need to make the triage test cost effective has been fulfilled by the public sector health care delivery system resulting in an edge over the private health system. This forms a founding basis of an effective and productive public private sector link.^9^ CAD4TB software system is not an alternative to clinical CXR reading but has an advantage to be used for mass screening with the ability to overcome intra and inter observer variability of human reading. This screening is based on chest radiograph symptom fusion.^10^

## METHODS

### Software details and target population:

CAR4TB software (Diagnostic Image Analysis Group, Radbound University Medical Centre, Nijmegen, The Netherlands) installed in a mobile x-ray system was used for screening of pulmonary TB in a high volume, tertiary care public sector hospital. The target population being individuals having high suspicion of pulmonary TB referred from the chest clinic.

### Study design and selection of input data:

This screening study was conducted by Radiology Department Medical Teaching Institute Lady Reading Hospital Peshawar, on data collected from screened individuals over a period of one year from April 2018 to April 2019 after ethical review board approval dated 1^st^ March 2018, with reference number 579/LRH/MTI. Data was collected from CAD4TB analysis of chest x rays of patients presenting with lower respiratory tract symptoms. Symptom for screening include cough lasting longer than two weeks, haemoptysis, weight loss, fever or night sweats. Individuals were screened by a mobile x-ray unit system equipped with a digital x-ray, printer and an online x-ray system supported by CAD4TB software. Individuals of both genders with an age range of 15 to 80 years were included in the study while children under the age of 14 years were excluded. This screening program was implemented in collaboration with the Indus Hospital Network under the umbrella of “Zero TB Project”. Informed consent was taken from all the participants of study.

### Chest X-ray scoring procedures:

**Fig.1:**
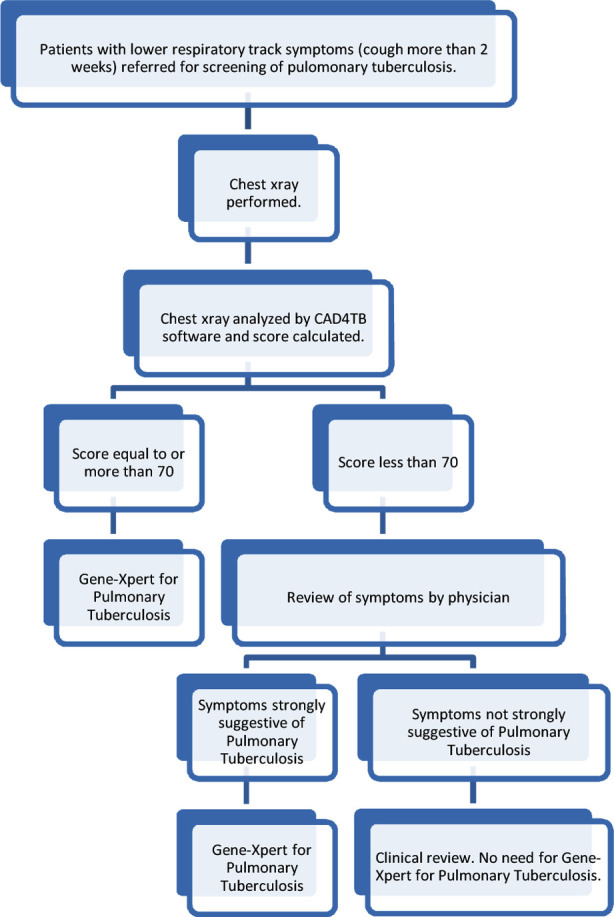
Tuberculosis testing algorithm for screening of patients with lower respiratory track symptoms.

The radiographs were analyzed by the CAD4TB software, developed using machine learning methods and sets of ‘training’ radiographs. It quantifies various image characteristics of a CXR to compute an ‘abnormality score’ that can take integer values ranging from 0 to 100.^8^ The analysis was based on detecting textural and shape abnormality in lung fields which are segmented automatically. Images with score of 70 and above were considered presumptive of tuberculosis while those individuals below the 70 score were reviewed clinically and selected cases considered presumptive on the basis of their symptomatology as presented in Fig.1. The threshold of 70 was chosen, in accordance with rational use of GeneXpert cartridges.^3^

### Data management and analysis:

The CAD4TB data was stored in a password protected software for patient confidentiality. The individuals referred to the TB center for Gene-Xpert analysis were registered online and allocated a unique patient ID. CAD4TB scores were tallied with patient age, gender, symptomatology and Gene-Xpert results. Statistical analysis was done as depicted in the tables and charts. Gene-Xpert is taken as the gold standard having sensitivity and specificity of 81.4 and 93.4 respectively,^5^ and comparable to Malik et al study.^11^ Total number of individuals screened for pulmonary TB by chest radiography were 26,997, and analyzed by the CAD4TB software, out of whom 2100 sputum samples were obtained for Gene-Xpert.

### Statistical analysis:

SPSS version 20 was used for statistical analysis of the data. Frequency and percentage were calculated for categorical variables like, GeneXpert, CAD4TB, and gender. Numerical variable mean ± SD were used and the data was post-stratified age wise. Sensitivity (True Positives x 100/ Total Diseased), specificity (True Negatives x 100/ Total Non-Diseased), negative predictive value (True Negatives/ True Negatives + False Negatives) and positive predictive value (True Positives/ True Positives + False Positives) were used for CAD4TB with Gene-Xpert as reference standard. Pie chart of gender distribution and Bar Graph of age variable stratification were also presented.

## RESULTS

Total number of individuals screened were 26,997, of which 18,811 (69.7%) were male and 8,186 (30.3%) females. 2,617 (9.7%) individuals were found presumptive for pulmonary TB either by CAD4TB on x-ray findings and symptomatology, and were advised Gene-Xpert. 24,380(90.7%) were nonpresumptive on both CAD4TB and symptomatology. Sputum samples for Gene-Xpert were obtained in 2,100 (80.24%) patients, as 517 (19.7%) patients were lost to follow up. Of the remaining, 1315 (62.6%) patients were male and 785 (37.4%) were female.

Out of the total of 2100 patients who underwent Gene-Xpert testing on sputum samples, 1,825 (86.9%) were presumptive for pulmonary TB on CAD4TB, Gene-Xpert was positive in 159 (8.7%) patients and negative in 1,666 (91.3%) individuals. In the remaining 275 patients, symptomatology was suggestive of possible TB, and 32 (11.6%) patients tested positive while 243 (88.3%) tested negative for Gene-Xpert. The sensitivity of CAD4TB score along with symptomatic review for tuberculosis with threshold score of 70 is 83.2% and specificity is 12.7%. PPV is 8.7%, NPV is 88.4% with 9.1% prevalence.

**Table I:** Diagnostic accuracy of CAD4TB score and symptomatic review for tuberculosis with threshold score of 70.

Presumptive TB on CXR on basis of CAD4TB Score and symptoms review with a cutoff of 70		Gene-Xpert Result		

		MTB Detected	MTB not Detected	
Yes	Count	159 True Positive	1666 False Positive	1825
	Percentage	83.2% SENSITIVITY	87.3%	86.9%
No	Count	32 False Negative	243 True Negative	275
	Percentage	16.8%	12.7% SPECIFICITY	13.1%
Total	Count	191	1909	2100

Age stratification was done from 15-75 years with the largest number of people screened in the 15-30 years age group and minimum screened in 61-75 years. Highest prevalence was in the 46-60 years (30.6%) followed by 15-30 years (22.2%) with the least prevalence in 76 and above (5.6%) on CAD4TB.

## DISCUSSION

Tuberculosis is a leading global health problem. Estimated 09 million emergent cases of TB and 1.5 million deaths occurred in 2013. In 2014, 9.6 million people developed TB and 1.5 million died from the disease, in 2015, 10.4 million new TB cases reported, but 6.1 million (59%) notified to National TB Program. A large fragment of the population remains undiagnosed due to lack of awareness and resource limitations. World Health Organization emphasizes proactive approach to early detection of tuberculosis to fulfill the target of global TB elimination. Of the 22 high burden countries accounting for over 80% of world’s TB cases, Pakistan ranks 5^th^.^12^

WHO’s screening for tuberculosis has 10 algorithms.^5^ Of the option’s available symptom screen and CXR are the most commonly employed. Symptom screening has a low sensitivity, especially in early TB but is cost-effective.

CXR is evidence based and definitive screening tool for TB because of its higher sensitivity. A study in South India on the yield of cases by different screening methods,^13^ showed that symptoms screening picked up about two-third of the cases, whereas chest x-ray alone picked up more than three-fourths of the cases.

CXR in addition is a valuable tool as it provides screening for medical conditions other than TB. However, CXR lacks specificity hence a significant proportion of individuals without TB will have an abnormal test result on CAD4TB. In our study the specificity was low due to limited use of gene Xpert catridges hence decrease in CXR is used with a bacteriological confirmatory test for tuberculosis which makes it an effective pre-screening tool, before the more expensive test (Gene-Xpert) can be carried out for diagnosis.^14^ Alternatively QuantiFeronTB-Gold test can be used an adjunct to diagnosis in patients having clinical and radiological data compatible with pulmonary tuberculosis.^15^

CAD4TB analyses the images to creates heat maps, on the basis of opacities in lung fields and gives it a particular abnormality score. In Fig.3, the first column is input CXR, and second column is heat map resulting from texture analysis, yielding a range of colours to indicate low-to-high suspicion of abnormality in the following order of blue-green-yellow-orange-red. The last column shows TB score assigned by CAD ranging from 0 (normal) to 100 (abnormal). CAD4TB analyses the images, hence promoting TB systematic screening, early case detection and triaging.^16^

**Fig.2:**
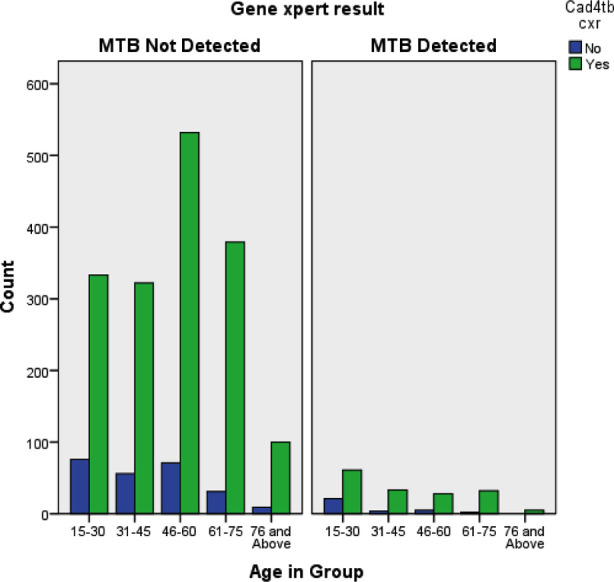
Bar chart of age variable stratification for gene expert analysis.

**Fig.3:**
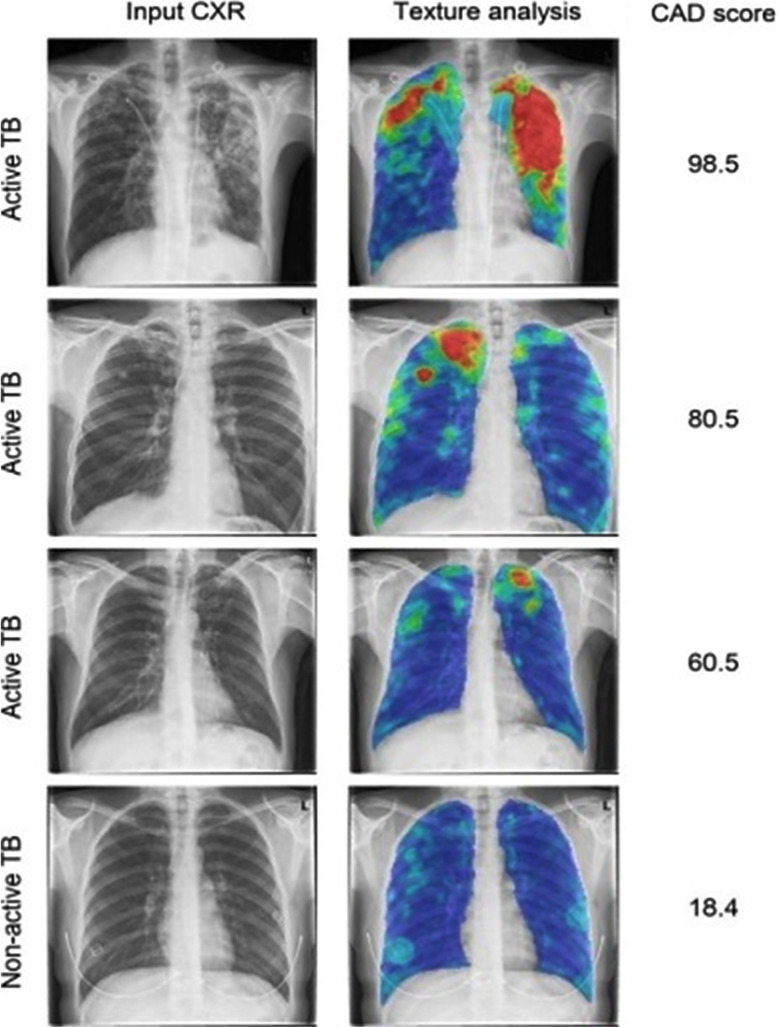
Pictorial Comparison of Input Chest Xray and CAD4TB Scores.

Comparison with study conducted by Zaidi et al.^7^ shows differences in the form of the size of the targeted population as our study was conducted on a larger population group in a public sector hospital while the study by Zaidi et al was conducted on filtered targeted population in private sector. Similar trends were noted in the form of increased prevalence in females as result of malnutrition and multiple child births with poor access to quality health care. Another similar study was conducted by Madhani et al.^3^ which shows strong association and supports the idea of implementation of CAD4TB scores and Xpert positivity in mass screening.

CXR is also used as a diagnostic and screening tool for non-tuberculous lung diseases.^17^ This could lead to the potential misdiagnosis or missing a more serious illness like a tumour. To implement CAD4TB solely for detecting TB, as triage tool is acceptable but for geneXpert negative cases, yet yielding abnormality on chest radiograph, this can be corrected by adding a human reader like radiologist, followed by physician review.

Active screening among household contacts is a documented effective way to improve TB case detection. New TB cases amongst contacts with abnormal x-ray was recorded by Dina et al^18^ and the use of Chest X-ray in combination with symptom screen was recommended.

The results of this study suggest that combination of CAR and symptomatology when used for screening of TB in a symptomatic population are not only reasonable effective algorithm but also more cost effective. Studies have evaluated and identified neighborhood contacts, and specifically those within 50m of an index case,^19^ to comprise a viable target population for intensified screening with productive yields.^20^

Three years of community-wide screening in persons 15 years of age or older who resided in Ca Mau Province, Vietnam, resulted in a lower prevalence of pulmonary tuberculosis in the fourth year than standard passive case detection alone.^21^ A study by Murphy et al^22^ compares different versions of CAD4TB software and recommends CAD4TB version 6 for optimal performance.

The future projects for this study would include research related to different risk factors association with TB and use of different cutoff values for CAD4TB software. In a recent study by Habib et al^23^ associated of diabetes mellitus and TB were assessed by using CAD4TB software. The strategy recommended by WHO for diagnosing pulmonary TB in a nationwide survey is symptom and chest X-ray screening, followed by smear microscopy and culture examinations for those with an abnormal X-ray and/or TB symptom’s and this is exactly the pattern followed in our study for early detection of tuberculosis in mass screening.^24^

### Limitations

- Short-term 12 months, single centre study.
- Data collected from individuals presenting to hospital with lower respiratory tract symptoms and may not be the true representation of disease burden in the population.
- Chest radiographs scored by CAD4TB were not independently analysed by trained Radiologist.
- Not all patients screened with chest radiographs analysed by CAD4TB had GeneXpert obtained, hence affecting specificity of the study.

## CONCLUSION

Computer Aided Detection for Tuberculosis software for Chest Xray analysis is a useful tool for mass screening of TB in high burden developing countries. It’s cost effective for screening and early active TB case finding in patients and their contacts, who would otherwise go undiagnosed.
